# Navigating the Complexities of Laryngeal Tuberculosis: A Comprehensive Case Report and Literature Review

**DOI:** 10.7759/cureus.46505

**Published:** 2023-10-04

**Authors:** Araya Gautam, Harendra Kumar, Abubakar Gapizov, Pratik Paudel, Rakshya Gautam

**Affiliations:** 1 School of Health Sciences, Teesside University, Middlesbrough, GBR; 2 Medicine and Surgery, Dow University of Health Sciences, Karachi, PAK; 3 General Surgery, American University of Antigua, Coolidge, ATG; 4 Neurology, Annapurna Neuro Hospital, Kathmandu, NPL; 5 Internal Medicine, Alka Hospital, Lalitpur, NPL

**Keywords:** voice disorders, tuberculosis, treatment, management, diagnosis, clinical presentation, navigating complexities, literature review, comprehensive case report, laryngeal tuberculosis

## Abstract

This case report and literature review presents a detailed exploration of the diagnosis and management of laryngeal tuberculosis, emphasizing the challenges encountered in dealing with rare and multifaceted medical conditions. Through a systematic analysis of the patient's clinical journey and an insightful review of pertinent literature, the study underscores the complexity inherent in diagnosing primary laryngeal tuberculosis and highlights the growing relevance of this rare extrapulmonary manifestation. The case showcases the significance of a comprehensive diagnostic approach, the collaboration of diverse medical specialists, adherence to established treatment guidelines, and the crucial role of continuous patient monitoring. The successful resolution of this intricate case serves as a compelling testament to the power of interdisciplinary coordination and precision medicine, providing valuable insights into navigating the intricate landscape of laryngeal tuberculosis.

## Introduction

Laryngeal tuberculosis (TB), a rare and neglected disease, has a misdiagnosis rate of up to 80% due to the lack of specific signs or symptoms [1]. Nevertheless, it has evolved to become the most prevalent granulomatous condition in endemic countries affecting the larynx, predominantly impacting men aged 40-60 years [1].

Several risk factors contribute to the likelihood of upper respiratory tract TB infection, including HIV infection, tobacco use, diabetes, recreational drug consumption, malignancies, and immunosuppressive drug utilization. In the realm of laryngeal infection, the disease primarily affects the anterior laryngeal structures, which are the parts of the larynx that are located in the front of the neck. They include the thyroid cartilage, the epiglottis, the vocal cords, and the vestibular folds materializing as hypertrophic, exophytic, or polypoid lesions [1,2]. The prevalence of hypertrophic, exophytic, or polypoid lesions in upper respiratory tract TB infection is not very well documented in the literature. However, some studies have reported the occurrence of these lesions in patients with laryngeal TB, which is a rare form of extrapulmonary TB that affects the anterior laryngeal structures. These lesions can cause symptoms such as hoarseness, dysphonia, dysphagia, odynophagia, and hemoptysis [1,2]

Vocal cords bear the brunt in 50-70% of cases, while false cords are implicated in 40-50% of cases. These critical anatomical regions are vulnerable to inflammation during severe infections, culminating in airway obstruction. The ensuing consequence is rapid respiratory distress, which can escalate to respiratory failure if not promptly identified and managed with appropriate measures [2,3].

The current case demonstrates the complicated diagnostic challenges presented by seemingly disparate symptoms and medical histories. The medical team successfully navigated these problems with a deliberate approach, interdisciplinary collaboration, and a commitment to evidence-based principles.

## Case presentation

A 24-year-old Filipino male presented with a chief complaint of difficulty breathing. Past medical history includes bronchial asthma. Family history reveals bronchial asthma in his mother and brother; his mother also had Pott's disease but had been refusing anti-TB treatment. The patient was a driver and egg dealer, residing in a bungalow-type dwelling with his wife, with a history of smoking (nine pack-years) and occasional alcohol consumption (one to two sessions per month). He did not chew betel nuts and used liquefied petroleum gas for cooking at home.

Two years prior to the presentation, he had experienced sudden onset epigastric discomfort with burning pain, which reached an intensity of 5/10 and ascended to his throat. This pain worsened during fasting but was relieved by food intake. He occasionally vomited non-bilious, non-bloody contents after meals. After a visit to a rural health center, he was diagnosed with "hyperacidity" and received unspecified medications that provided temporary relief. Over the following months, he endured persistent epigastric pain but did not seek medical attention. Seven months prior, he developed intermittent, rough, breathy hoarseness that worsened with exertion but improved with voice rest. A private ENT physician diagnosed laryngopharyngeal reflux, and treatment with omeprazole and an unrecalled nasal spray offered temporary relief. As the hoarseness persisted, it led to another ENT consultation, which revealed a suspicious laryngeal mass.

Despite a recommended laryngeal CT scan, the patient's symptoms improved, but he continued to experience hoarseness. Additionally, he began to experience episodes of noisy, wheezy breathing without apparent triggers, requiring salbutamol nebulization at a local hospital. Three weeks prior to presentation, the frequency of hoarseness and breathing difficulties increased, prompting a visit to a district hospital, where he was evaluated as a probable pulmonary (PTB) suspect, with negative direct sputum smear microscopy (DSSM) results and no initiation of anti-TB medications. Shortly before admission, he developed sudden dyspnea, leading to initial evaluation by the Internal Medicine service, which attempted but failed emergency intubation. Subsequently, he was referred to anesthesiology for a challenging intubation and to the ENT service for possible emergency tracheostomy, resulting in his admission.

**Figure 1:**
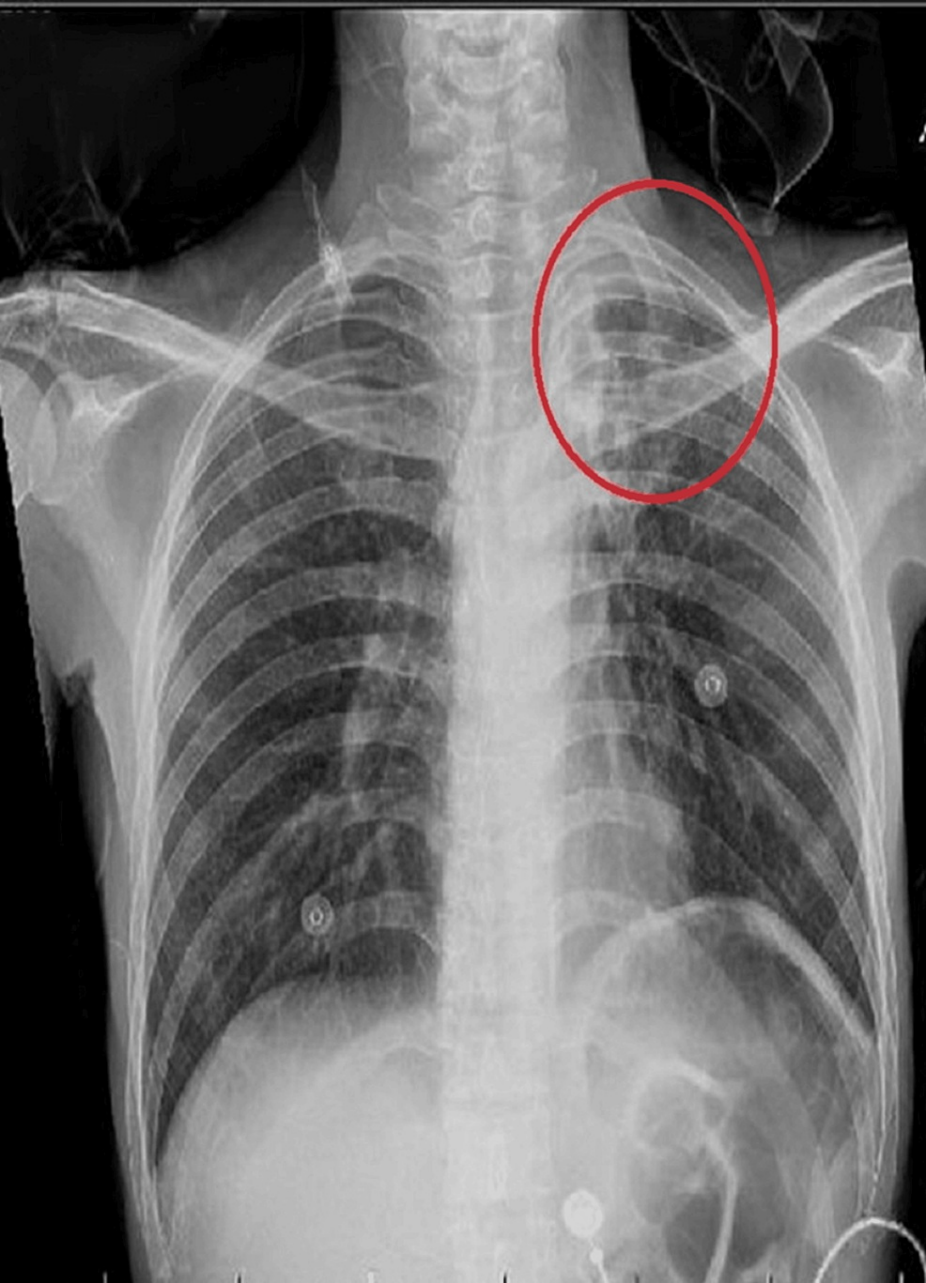
Chest X-ray showing reticulo-nodular opacities in the left upper lobe with apical pleural thickening, volume loss and upward traction of the hilum

In pursuit of an accurate diagnosis, a systematic approach was adopted. Traumatic causes, inhalation injury, neoplasms, and oropharyngeal obstruction were ruled out through detailed clinical examination and laryngoscopic findings. Table 1 shows the complete blood count and differential count results. The initial chest X-ray as shown in Figure 1 revealed bilateral hilar lymphadenopathy and normal lung parenchyma. Comprehensive imaging, including chest and neck CT, displayed extensive mediastinal lymphadenopathy encasing the trachea, suggesting impending upper airway obstruction.

**Table 1:** Complete blood count and differential count results MCV: mean corpuscular volume; MCH: mean corpuscular hemoglobin; MCHC: mean corpuscular hemoglobin concentration

Test	Result	Unit	Reference Range	Status
Complete Blood Count				
WBC	26.98	10e3/uL	3.70-11.3	High
RBC	4.64	10e6/uL	4.52-5.90	
Hemoglobin	142	g/L	125-175	
Hematocrit	0.396	L/L	0.370-0.113	
MCV	85.3	fL	77.0-95.9	
MCH	30.7	Pg	27.4-32.5	
MCHC	35.7	g/dL	32.0-34.8	High
Platelet	210	10e3/uL	149-451	
Differential Count				
Neutrophil	97.5	%	37.0-80.0	High
Lymphocytes	1.6	%	10.0-50.0	Low
Monocytes	0.8	%	0.00-0.12	
Eosinophils	0	%	0.00-7.00	
Basophils	0.1	%	0.00-2.50	

**Figure 2:**
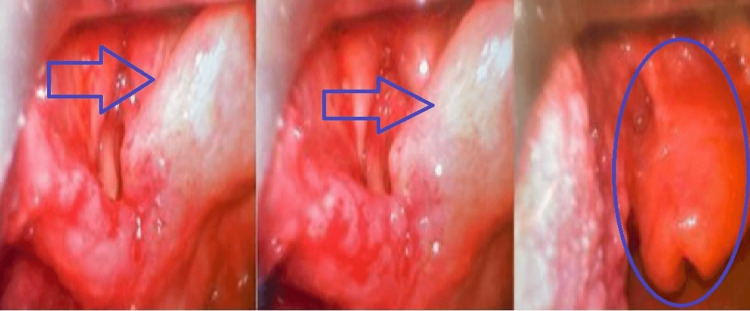
Flexible laryngoscopy revealed hyperemia and edema of the epiglottis, arytenoids true and false vocal cords, moth-eaten appearance, pooling of saliva, and narrowed rima glottidis

Laryngoscopy as shown in Figure 2 revealed diffuse edema, erythema, ulcers, and immobility of vocal cords, consistent with TB laryngitis, a type of extrapulmonary TB affecting the larynx. The pathophysiology of laryngeal TB was discussed, focusing on *Mycobacterium tuberculosis* transmission through inhalation and subsequent inflammation causing edema, ulcers, and vocal cord immobility. Mediastinal lymphadenopathy's impact on the recurrent laryngeal nerve and cricoarytenoid joint's implications were emphasized.

Airway security was paramount due to potential complications of laryngeal TB. Immediate measures, including tracheal intubation, were taken to ensure respiratory support in case of airway obstruction. A multidisciplinary team, comprising pulmonologists and infectious disease specialists, collaborated in patient care. Following the National TB Program guidelines for extrapulmonary TB, the patient received a four-drug regimen: isoniazid, rifampicin, pyrazinamide, and ethambutol. Liver function and side effects were closely monitored. During hospitalization, the patient's laryngeal edema and respiratory symptoms improved significantly. After two months of anti-TB treatment, repeat laryngoscopy demonstrated regression of ulcerations and improved vocal cord mobility. The patient completed the six-month anti-TB treatment without adverse effects. Six-month and one-year follow-up appointments post-treatment revealed sustained improvement, and the patient remained asymptomatic throughout this period.

## Discussion

*Mycobacterium tuberculosis* causes TB, an infectious disease characterized by granulomas [1,2,4]. While it mostly affects the lungs, resulting in PTB, it can also affect extrapulmonary areas such as the larynx [5]. This disease is a significant public health concern in developing countries, particularly in the lungs, which account for 80% of cases [4,6]. Nonetheless, it can cause damage to any part of the body.

Primary laryngeal TB is an uncommon kind of extrapulmonary TB that accounts for fewer than 1% of all TB cases [4,6,7]. However, due to a rise in the number of immunocompromised individuals and the prevalence of multidrug-resistant TB, cases of laryngeal TB have become increasingly common [7]. Extrapulmonary TB accounts for around 30-40% of cases and may affect a variety of organs including eyes, ears, heart, and adrenal glands [4,6,7]. TB may affect the larynx, despite the fact that it is seldom addressed. Due to laryngeal inflammation and granulations, laryngeal TB may cause a range of symptoms, from slight voice hoarseness to severe discomfort while swallowing (odynophagia) and difficulty breathing (dyspnea) [8,9]. Given the global resurgence of TB, which is being fueled in part by the spread of HIV, healthcare practitioners and ENT specialists should be aware of the possibility of TB in the larynx [9-11].

While laryngeal TB is rare, it presents a difficult diagnostic challenge due to symptoms that are similar to those of many other respiratory disorders. Early symptoms such as inspiratory stridor, dyspnea, and hoarseness might have been readily attributed to more common illnesses such as vocal cord dysfunction, laryngopharyngeal reflux, or even obstructive sleep apnea [7,9,12]. His medical history, however, painted a more convoluted picture, with a history of bronchial asthma, a family history of Pott’s disease, and smoking habits.

The presence of constitutional symptoms such as fatigue, weight loss, and nocturnal sweats complicated the diagnosis even further. A focused technique proved critical in traversing this thick maze of symptoms. After ruling out traumatic causes, inhalation injuries, neoplasms, and oropharyngeal obstructions, the diagnostic range was narrowed to TB-related disease. The presence of bilateral hilar lymphadenopathy on the initial chest X-ray served as a significant clue, shifting the focus of the diagnosis to possible mediastinal involvement. Following extensive imaging, including chest and neck CT scans, substantial mediastinal lymphadenopathy encasing the trachea was seen, raising concerns about imminent upper airway obstruction. Laryngoscopy confirmed the diagnosis, revealing typical TB laryngitis symptoms such as widespread edema, erythema, ulceration, and vocal cord immobility.

With the possibility of an impending airway obstruction, maintaining the patient’s airway security became a top concern. Swift and urgent actions, such as tracheal intubation and standby surgical airway management, were critical in preventing respiratory distress. The collaborative efforts of pulmonologists and infectious disease specialists were critical in developing a comprehensive and effective treatment approach. This regimen, which included isoniazid, rifampicin, pyrazinamide, and ethambutol, not only targeted the causative agent *Mycobacterium tuberculosis* but also aimed to reduce the development of drug resistance. As the foundation of the treatment plan, rigorous and consistent monitoring of liver function and potential side effects was devised, ensuring patient safety and drug adherence.

Investigating the pathophysiology of laryngeal TB unveils the intricate interplay of the immune system's reactions. Inhaling *Mycobacterium tuberculosis* causes an inflammatory cascade that results in vocal cord swelling, ulceration, and eventual paralysis [3,11,12]. The addition of mediastinal lymphadenopathy adds another layer of complexity, potentially jeopardizing critical structures including the recurrent laryngeal nerve and the cricoarytenoid joint.

The trajectory of the patient's clinical growth throughout the treatment journey attests to the management technique's efficacy. There were noticeable reductions in laryngeal edema and respiratory symptoms two months after starting the anti-TB regimen. A second laryngoscopy revealed ulcer regression and significant improvement in vocal cord mobility, confirming the effectiveness of the chosen treatment approach.

## Conclusions

This report provides a comprehensive examination of the diagnosis and treatment of laryngeal TB, shedding insight into the complexities associated with unusual and different medical conditions. The current case exemplifies the difficulties in diagnosing primary laryngeal TB and demonstrates the growing relevance of this uncommon extrapulmonary presentation. It emphasizes the need for a thorough diagnostic approach, collaboration among many medical specialists, adherence to established treatment procedures, and the critical significance of continued patient monitoring. The successful resolution of this tough case is a dramatic testament to the efficacy of multidisciplinary collaboration and precision medicine, providing critical insights for negotiating the complex environment of laryngeal TB. The case highlights the need to take a logical and collaborative approach when dealing with such complex medical concerns, which finally leads to a positive outcome for the patient.
